# Supplementary figures and images for: Temporal Quantitative Proteomic and Phosphoproteomic Profiling of SH-SY5Y and IMR-32 Neuroblastoma Cells during All-Trans-Retinoic Acid-Induced Neuronal Differentiation
Source: Int J Mol Sci. 2024 Jan 15;25(2):1047. doi: 10.3390/ijms25021047 (PMC10816102; doi:10.3390/ijms25021047)

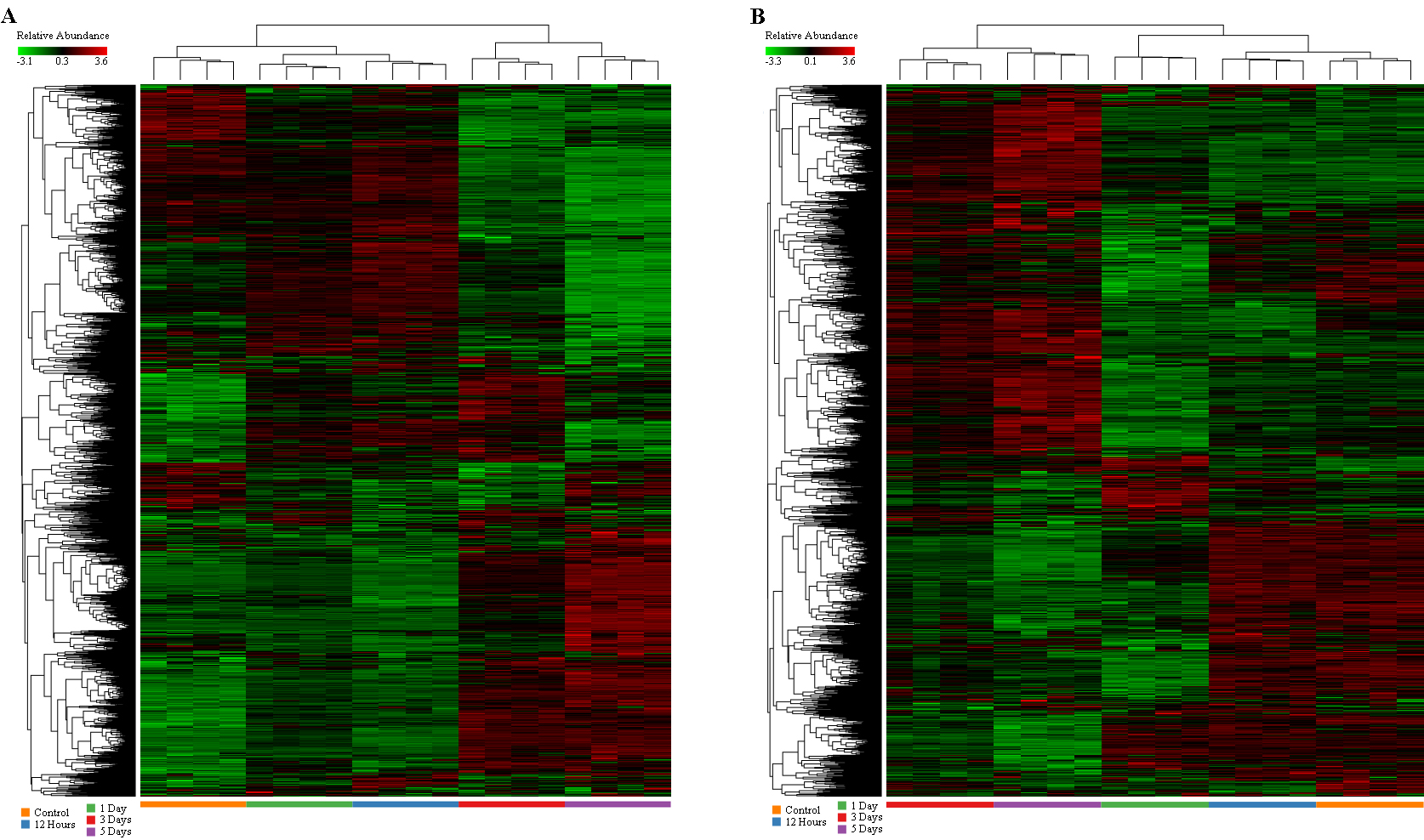

Supplement: Supplementary file 1 [file ijms-25-01047-s001.zip › ijms-2751594-supplementary/Supplementary Materials/Supplementary Figure S3.jpg]

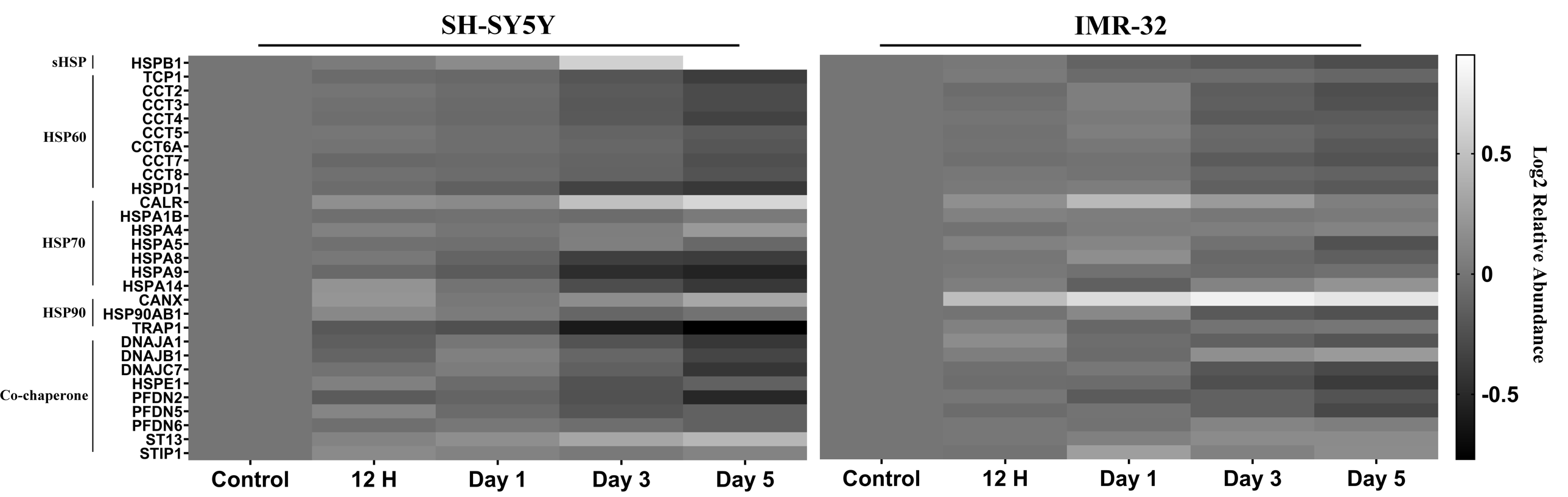

Supplement: Supplementary file 1 [file ijms-25-01047-s001.zip › ijms-2751594-supplementary/Supplementary Materials/Supplementary Figure S5.jpg]
